# Sentinel lymph node biopsy in esophageal cancer: an essential step towards individualized care

**DOI:** 10.1186/1750-1164-8-2

**Published:** 2014-05-05

**Authors:** George L Balalis, Sarah K Thompson

**Affiliations:** 1 Department of Surgery, Level 5, Eleanor Harrald Building, Royal Adelaide Hospital, Adelaide, South Australia 5000, Australia

**Keywords:** Sentinel lymph node, Esophageal cancer, Micrometastasis, Isolated tumor cell, Endoscopic mucosal resection

## Abstract

Lymph node status is the most important prognostic factor in esophageal cancer. Through improved detection of lymph node metastases, using the sentinel lymph node concept, accurate staging and more tailored therapy may be achieved. This review article outlines two principle ways in which the sentinel lymph node concept could dramatically influence current standard of care for patients with esophageal cancer. We discuss three limitations to universal acceptance of the technique, and propose next steps for increasing enthusiasm amongst physicians and surgeons including the development of a universal tracer, and improved contrast agents with novel dual-modality ‘visibility’.

## Introduction

In the United Kingdom, the rate of death from esophageal cancer in men has increased by more than 65% since the 1970’s [1,2]. Over the years, there has been some improvement in treatment outcomes, with neoadjuvant therapies and better patient selection [3,4]. There is still however much room for improvement, as current survival rates for resectable disease remain less than 50% at five years.

Regional lymph node status is the single most important prognostic factor for patients with esophageal cancer [5]. It is this prognostic factor that has spawned considerable interest in the sentinel lymph node (SLN) concept, as a method for decreasing the extent of surgery, as well as improving staging of patients, by concentrating the pathologist’s attention on 1 or 2 important lymph nodes.

The sentinel lymph node (SLN) concept, first described by Morton in the early 1990s, depicts the preferential lymphatic metastasis of a tumor to one or more regional nodes. It is the gold standard for patients with breast cancer and malignant melanoma [6-10]. The ALMANAC trial, demonstrated a marked reduction in morbidity and mortality associated with SLN biopsy compared to routine axillary lymphadenectomy, in patients with breast cancer [11,12]. This has been further demonstrated in several meta-analyses and randomised control trials [13]. In melanoma patients, it allows 80% of patients to be spared a formal lymph node dissection, avoiding the complications of lymphadenectomy; post-operative infection, seroma, long-term stiffness, sensory changes in a peripheral limb dissection, and most importantly, lymphedema [14,15]. Due to its low false-negative rate, quoted at around 1%, SLN biopsy negative patients can be assumed to have no microscopic disease in the remainder of the lymphatic basin [14,16].

So why has the SLN biopsy not become standard of care in esophageal cancer, and are there similar benefits to be had?

## Review

### Benefits

There are two principal ways in which routine SLN biopsy in patients with esophageal cancer could dramatically influence current treatment options.

### Endoscopic Mucosal Resection/Endoscopic Submucosal Resection (EMR/ESR)

Similar to breast cancer and melanoma patients, more accurate preoperative SLN detection could improve the ability to tailor resection of a more superficial esophageal cancer, and potentially avoid the need for an esophagectomy. The risk of nodal disease in pT1a (intra-mucosal) lesions has been shown in most studies to be less than 5%, compared to 12 to 37% in pT1b (submucosal) lesions [17-20]. A recent study by Manner *et al.* describes successful ESR of esophageal cancers restricted to the upper third of the submucosa (pT1b sm1 lesions) in 66 patients over a 15-year time period [21]. They achieved an 87% complete endoluminal remission rate and, in patients with small focal lesions less than 2cm in size, a 97% complete remission rate. In this paper, they started with double the number of patients, but excluded those with high-risk features for lymph node metastasis. If we could preoperatively assess lymph node involvement with precision, up to 88% of pT1b sm1 patients could avoid a highly morbid esophagectomy, and instead undergo a much less invasive ESR. At present however, Sepesi *et al.*, have concluded that superficial submucosal esophageal adenocarcinoma should not be treated by endoscopic resection alone, until more accurate predictors of nodal spread are found [22].

Currently, there is no randomized controlled trial, which compares EMR or ESR to esophagectomy for early esophageal cancer (pT1). Various retrospective analyses show that EMR/ESR is comparable to esophagectomy, with similar complete remission rates and 5-year overall survival. EMR/ESR has also been shown to result in less morbidity and mortality [23-25]. These studies however suffer from their retrospective nature, heterogeneity in patient groups, and heterogeneity in treatment modalities. It appears that, at present, pT1a esophageal cancer can be treated quite safely with EMR alone, however pT1b cancers warrant a more invasive esophagectomy and lymphadenectomy until we can improve on current preoperative investigations [26].

### Histopathological assessment

Selective identification of the most important lymph nodes allows the pathologist to "ultra stage" these nodes with serial sectioning, immunohistochemistry (IHC), and/or reverse-transcriptase polymerase chain reaction (RT-PCR). In an ideal world, all resected lymph nodes would undergo this rigorous assessment. However routine serial sectioning and IHC is prohibitively expensive and time consuming, and therefore a more selective approach are required to ensure that only the most important nodes are selected for the pathologist.

Why is such a detailed pathological assessment necessary? The 7th Edition of AJCC Cancer Staging Manual upstages patients with a breast cancer ≤20 mm with nodal micrometastases only from Stage 1A to Stage 1B, to reflect a poorer outcome [27]. Accordingly, nodal micrometastases are classified pN1mi, and not pN0mi. At the current time however, isolated tumor cells (ITCs), are still considered pN0 disease even though a New England Journal of Medicine paper, published in 2009, found that patients with favorable early-stage breast cancer and either micrometastases or ITCs in regional lymph nodes had a reduced 5-year rate of disease-free survival [28].

In esophageal cancer, even in patients with pT1N0M0 disease, micrometastases are associated with a significant negative impact on survival [29-33]. These occult deposits, either micrometastases or isolated tumor cells, are not visible using conventional pathology and require both serial sectioning and IHC. Further to this, Thompson *et al.* identified that isolated tumor cells are as important as micro-metastases in determining the overall survival of patients with esophageal cancer [34]. Yonemura *et al.* also found that a larger proportion of patients died from recurrent disease, if found to have isolated tumor cells [35]. This clearly has important implications for accurate staging and tailoring therapy, in patients with esophageal cancer.

### Current Limitations

First, and probably most important, the type of radiocolloid available for clinical use to detect sentinel nodes is strongly dependent on that particular country’s legislation, which hinders the development of uniform protocols. A radiocolloid should "show rapid transit towards sentinel nodes with persistent retention in the nodes" [36]. The balance of these two properties lies in the size of the particle [37]. Smaller particles allow faster visualization of SLN and better uptake in metastatic nodes, whilst larger particles have the advantage of a longer retention time and slower transit, which minimizes detection of nodes downstream to the SLN.

Throughout Europe, ^99m^Tc-albumin is used, compared to ^99m^Tc-tin fluoride colloid in Japan, ^99m^Tc-sulfur colloid in North America, and ^99m^Tc-antimony colloid here in Australia. The size of the colloid ranges from a 100-220nm sulphur, which can be injected one day prior to surgery, to 10 ± 3 nm antimony that is injected just prior to the operation [38,39]. Smaller sized colloids were found to have greater success in preoperative visualization and intraoperative identification of axillary sentinel nodes in breast cancer patients, compared to larger sized colloids [40]. No similar studies exist for upper gastrointestinal cancer patients.

A second limitation, alluded to earlier, is the logistical issue of injecting the colloid. This is a simple superficial injection in melanoma and breast cancer patients. In contrast, the tracer must be injected via endoscopy/colonoscopy or laparoscopy/thoracoscopy in patients with a cancer of the gastrointestinal tract. This is much more invasive than a skin injection and the timing differs depending on the radiotracer legislated for use in the country of origin.

Third, in oesophageal adenocarcinoma, greater than 95% of lymph nodes are within 3 cm of the primary tumor, as demonstrated by van de Ven *et al*. [41]. This complicates the detection of lymph nodes preoperatively by lymphoscintigraphy, with PET/CT scanners often unable to distinguish positive nodes close to the primary tumor due to the shine-through effect [38]: "where a strong radioactive signal from the primary tumor hinders the SLN detection with radiocolloid." The inability to have a clear anatomical pathway preoperatively, to guide the intraoperative dissection, is a deterrent towards routine clinical application of the SLN concept. The incorporation of more spatially accurate imaging modalities, and better minimally invasive gamma probes (i.e. with orthogonal 90-degree probes) may avoid interference from injected tracer in the primary tumor.

### Next steps

As stated above, esophageal cancer should adopt the SLN concept. It is the only practical method in today’s economic climate to identify the most important nodes for detailed histopathological analysis. As well, widespread adoption of this technique will promote the development of novel sentinel node tracers, which may even be capable of non-invasive lymph node staging, and delivery of therapeutic agents to disseminated tumor cells within the nodes. However, as an initial step, we need to improve upon detection of the SLN in non-superficial cancers.

Second, a universally appropriate tracer is required. This will provide consensus on methodology, timing of migration, and results of SLN detection. The timing of endoscopic injection will therefore be able to be standardized. Probably, a tracer with a longer half-life between injection and migration to sentinel nodes will be more appropriate, to enable preoperative imaging.

Third, improved contrast agents are needed, especially for esophageal cancer. An ideal tracer is one that can enter the initial lymphatic capillary with ease, and move freely to the SLN where it is retained. The tracer should be chemically stable, inexpensive, easily produced and reproducible, and concentrate in the node without spillage. It should also have a short transit time, but remain in the sentinel nodes to allow detection prior to moving on second-tier nodes. Nanotechnology, the use of man-made objects, which contain nano-scale dimensions, may provide the answer [42]. These particles have many of the properties listed above, and some, such as supramagnetic iron oxide nanoparticles (SPIONs), have already been approved for *in vivo* imaging. For example, in 16 patients with esophageal cancer, Nishimura *et al.* found that ferumoxtran-10 (an ultra-small 20 nm SPION) provided a combined accuracy of 96%, with 100% sensitivity and 95% specificity in locating the SLN [43]. Taking this application one step further, Weissleder *et al.* have shown that lymphotrophic SPIONs, injected systemically as exogenous contrast, can discriminate healthy versus tumor-burdened nodes by the degree of accumulation of particles in the nodes [44].

Dual-modality tracers using blue dye and radioisotope tracer have proven very reliable in many solid organ tumours, including breast cancer, melanoma, and gastric cancer [45-47]. This technique facilitates non-invasive preoperative imaging, coupled with subsequent intraoperative assessment, for SLN detection. Blue dye however is of limited use in esophageal cancer, due to its short transit time (too short when considering the amount of time needed to enter the chest cavity), and discoloration of adjacent tissues, which may obscure the surgical field. Different dual-modality tracers are needed; perhaps those using magnetic resonance imaging (MRI) with nanoparticles. MRI has advantages over CT lymphography as it provides higher spatial and temporal resolution, and avoids ionizing radiation. We are currently trialing a dual-modality tracer with both magnetic resonance imaging capability (using iron oxide) and radioactive properties (using ^99m^Tc-antimony colloid) in a pig model.

## Conclusions

There are two principle ways in which the sentinel lymph node (SLN) concept could improve staging and thereby individualized care in patients with esophageal cancer. First, adoption of the sentinel node biopsy as a staging investigation (i.e. preoperative lymph node assessment) could tailor resection of a more superficial esophageal cancer, and potentially avoid the need for an esophagectomy. Second, identification of the most important lymph nodes allows the pathologist to "ultra stage" these nodes with serial sectioning, immunohistochemistry, and/or reverse transcriptase polymerase chain reaction. This enables detection of micrometastatic disease, and identifies a patient subset that may benefit from adjuvant therapy.

Three limitations to widespread acceptance of the SLN concept in all solid organ tumors include the lack of a universally legislated radiocolloid for clinical use, the need for an invasive procedure to inject the colloid in many non-superficial cancers, and the inability to have a clear anatomical pathway preoperatively (due to the "shine through effect"), to guide intraoperative dissection.

We believe that esophageal cancers should adopt the SLN concept. This will promote intense research in this field, and lead to improved tracers capable of not only non-invasive lymph node staging, but delivery of therapeutic agents to disseminated tumor cells within the nodes. A universally appropriate tracer is also required to provide consensus and standardization on methodology, timing of migration, and results of SLN detection. Finally, improved contrast agents are needed, especially those with novel dual-modality ‘visibility’.

## Competing interests

The authors declare that they have no competing interests.

## Authors’ contributions

GB participated in the concept and design of the study, acquisition of data (comprehensive literature review), and drafting the manuscript. ST conceived of the study, and participated in its design. ST also revised the manuscript draft. Both authors read and approved the final manuscript.

## Authors’ information

GB has begun a Masters of Philosophy (Surgery) degree at the University of Adelaide, looking at improving detection of sentinel lymph nodes in oesophageal cancer, through the use of nanotechnology. He began General Surgery training, in South Australia, in 2013, with a long-term interest in upper gastrointestinal surgery. GB was appointed the Royal Australian College of Surgeons Trainee Representative for South Australia/Northern Territory (RACSTA) in 2013, with an interest in the continued improvement of surgical training.

ST was appointed as an Upper Gastrointestinal Surgeon at the Royal Adelaide Hospital in 2007. She completed her surgical training in Calgary, Canada in 2004, and subsequently completed a Minimally Invasive Surgery fellowship at the Oregon Health and Science University in Portland, Oregon, U.S.A., and an Upper Gastrointestinal Surgery fellowship in Adelaide, South Australia. ST completed a PhD in 2011 evaluating better diagnostic and staging tools for oesophageal cancer through ongoing work with sentinel lymph nodes. She was awarded an Associate Professor title in 2013 with the University of South Australia for her academic accomplishments, and is regularly invited to speak at National and International Scientific Meetings. She has authored more than 60 peer-reviewed scientific articles, and included as a chief investigator in obtaining over $1.4 million dollars in research and clinical development grants. She leads the research team within the Professorial Oesophago-Gastric Unit at the Royal Adelaide Hospital, and has clinical interests in gastro-oesophageal reflux disease and upper gastrointestinal cancer.
